# TREM1–NLRP3–driven pyroptosis in sepsis-associated acute kidney injury (AKI) with parallel autophagy changes

**DOI:** 10.1038/s41598-026-40893-w

**Published:** 2026-03-19

**Authors:** Xudong Liu, Qian Chen, Xiangyu Chen, Yaqi Wang, Junping Fan, Xinlun Tian, Xinjie Xu, Longxiang Su

**Affiliations:** 1 National Infrastructures for Translational Medicine, State Key Laboratory for Complex, Severe, and Rare Diseases, Institute of Clinical Medicine, Peking Union Medical College Hospital, Chinese Academy of Medical Science and Peking Union Medical College, Beijing, 100730 China; 2 Administrative Staff, Peking Union Medical College Hospital, Chinese Academy of Medical Science and Peking Union Medical College, Beijing, 100730 China; 3 Department of Critical Care Medicine, State Key Laboratory of Complex Severe and Rare Diseases, Peking Union Medical College Hospital, Chinese Academy of Medical Science and Peking Union Medical College, Beijing, 100730 China; 4 Department of Pulmonary and Critical Care Medicine, State Key Laboratory of Common Mechanism Research for Major Diseases, Peking Union Medical College Hospital, Chinese Academy of Medical Sciences & Peking Union Medical College, Beijing, 100730 China; 5 Department of Anesthesia, Critical Care and Pain Medicine, Center for Inflammation Research, Beth Israel Deaconess Medical Center, Harvard Medical School, Boston, 02215 USA

**Keywords:** Triggering receptor expressed on myeloid cells-1 (TREM-1), NLRP3 inflammasome, pyroptosis, autophagy, sepsis-associated acute kidney injury, Diseases, Immunology, Nephrology

## Abstract

Sepsis-associated acute kidney injury (SAKI) is a leading cause of critical illness and death, yet its upstream drivers remain unclear. Triggering receptor expressed on myeloid cells-1 (TREM-1) amplifies innate immune signaling. We investigated whether TREM-1 acts upstream of the NLRP3 inflammasome to promote pyroptotic injury in septic kidneys, while documenting accompanying changes in autophagy. Male mice with genetic deletion of *Trem1* and wild-type littermates underwent cecal ligation and puncture; kidneys and serum were collected at 24 h. A subset received pharmacologic modulation of the NLRP3 inflammasome (MCC950 inhibitor or nigericin activator). Renal injury was assessed by histology and tubular injury scores, serum creatinine and blood urea nitrogen, and cytokines. Inflammasome and pyroptosis readouts (NLRP3, cleaved caspase-1, cleaved gasdermin D, interleukin-1β) and autophagy markers (LC3B, Beclin-1, Atg5) were measured by Western blot and enzyme-linked immunosorbent assay. In human proximal tubular epithelial cells (HK-2), TREM-1 was overexpressed or silenced and cells were stimulated with lipopolysaccharide; the same readouts were quantified. *Trem1* deletion reduced kidney injury after sepsis, with lower tubular injury scores, improved serum creatinine and blood urea nitrogen, and decreased circulating cytokines. Renal inflammasome–pyroptosis activity was blunted in *Trem1*-deficient mice, with reduced NLRP3, cleaved caspase-1, cleaved gasdermin D, and interleukin-1β. Pharmacologic modulation aligned with this axis: MCC950 lessened injury in wild-type mice, whereas nigericin aggravated injury despite *Trem1* deficiency. In tubular cells, TREM-1 overexpression increased NLRP3 activation, interleukin-1β release, and pyroptosis markers, while TREM-1 knockdown produced the opposite pattern. Across models, *Trem1* loss or inhibition was associated with higher autophagy markers. Limited to the autophagy non-perturbed experimentally, these increases are interpreted as concomitant rather than causal. TREM-1 functions as an upstream amplifier of NLRP3 inflammasome activation and pyroptotic tubular injury in SAKI. Autophagy marker changes accompanied TREM1 loss; their functional relevance to NLRP3 activation remains to be determined. These data support soluble TREM-1 as a candidate early biomarker and nominate TREM-1 as a therapeutic target. Future work should define time courses, test post-insult TREM-1 blockade, and link circulating or urinary TREM-1 with renal inflammasome signatures to refine risk stratification and guide intervention.

**Supplementary Information:**

The online version contains supplementary material available at 10.1038/s41598-026-40893-w.

## Introduction

Sepsis remains a major global health challenge, affecting approximately 49 million people annually and causing nearly 11 million deaths worldwide. The kidney is often an early target in sepsis and may precipitate downstream organ dysfunction^1^. Acute kidney injury (AKI) is one of the most frequent and devastating complications of sepsis, occurring in 30–50% of hospitalized septic patients and in up to 60–70% of those admitted to intensive care units. Collectively, these figures correspond to an estimated 15–20 million cases of sepsis-associated AKI each year, highlighting an enormous public health burden and the critical need for improved mechanistic understanding, early biomarkers, and targeted interventions^2,3^. Accordingly, early identification of sepsis-associated AKI (SAKI) and mechanistic insight are crucial to guide therapy and improve outcomes. Sepsis reflects cascade amplification of innate immune and inflammatory responses. Beyond metabolic and excretory roles, the kidney, including the tubular epithelium, also performs endocrine and immunologic functions, positioning it centrally in sepsis-induced organ failure. Multiple studies indicate that inflammation is integral to the onset and progression of both sepsis and AKI, with complex bidirectional crosstalk between systemic and intrarenal pathways^4^.

Triggering receptor expressed on myeloid cells-1 (TREM-1) is an immunoreceptor predominantly expressed on monocytes/macrophages and neutrophils. Upon engagement by endogenous ligands or bacterial lipopolysaccharide, TREM-1 signals through the adaptor DAP12 to amplify innate inflammatory cascade^5^. The soluble form (sTREM-1) rises in body fluids during infection and has been widely evaluated as an early diagnostic biomarker^6^. Beyond classical immune cells, accumulating data indicate TREM-1 activity in parenchymal tissues, suggesting broader roles in organ injury^7^. We previously reported that urinary sTREM-1 identifies SAKI at an early stage^8^. and subsequent clinical studies have independently validated this association^9,10^. Importantly, sTREM-1 should be viewed as complementary to established AKI biomarkers rather than a replacement. Whereas NGAL and KIM-1 primarily report tubular stress or epithelial injury and creatinine/urine output reflect functional decline, sTREM-1 captures activation of an upstream innate immune amplification pathway. Thus, its potential advantage lies in pathway specificity and patient endotyping (i.e., identifying an “immune-amplified” sepsis AKI phenotype), while a key limitation is lower kidney specificity and susceptibility to systemic infection burden. This conceptual complementarity supports multi-marker strategies in which sTREM-1 is paired with injury-based biomarkers to refine risk stratification and guide mechanism-targeted intervention^11^. In parallel, clinical evaluation of the TREM-1 modulator nangibotide has shown acceptable safety; although the phase 2b ASTONISH trial did not meet its primary endpoint overall, exploratory and enrichment analyses indicate greater SOFA improvement at higher baseline sTREM-1 and a significant ΔSOFA benefit in patients with sTREM-1 ≥ 1050 pg/mL, collectively raising the prospect of kidney-relevant benefit in biomarker-selected sepsis^12^. Nevertheless, how TREM-1 activation mechanistically links to tubular injury in SAKI remains insufficiently defined, motivating the present study.

The pathological hallmarks of AKI include tubular epithelial injury, inflammatory signaling, and microvascular dysfunction. Injury and loss of renal tubular epithelial cells are central drivers of AKI. Multiple regulated cell-death programs contribute to tubular damage, notably apoptosis and pyroptosis, whereas autophagy is a conserved stress-adaptive process that modulates cell survival and injury. Under physiological and pathological conditions, autophagy is essential for renal homeostasis; basal autophagy in the kidney—particularly in the proximal tubule—is required for normal function and can limit endotoxemic AKI while reshaping tubular cytokine responses^13–15^. In our prior studies, TREM-1 activation under lipopolysaccharide (LPS) exposure increased tubular apoptosis and suppressed autophagy, whereas TREM-1 blockade reduced apoptosis and restored autophagy, in part via NF-κB–dependent signaling^16^. Pyroptosis is an inflammatory form of programmed cell death in which caspase-1 drives maturation of interleukin-1β and interleukin-18, accompanied by gasdermin-mediated membrane permeabilization and pro-inflammatory mediator release^17^. TREM-1, through the adaptor DAP12, amplifies upstream danger signals and can potentiate innate immune activation within the kidney microenvironment.Among inflammasome pathways relevant to sepsis and organ injury, NLRP3 integrates pathogen- and damage-associated cues to elevate caspase-1 activity and inflammatory cytokine release, thereby linking innate sensing to tubular injury.

We hypothesized that TREM-1 drives NLRP3 inflammasome activation while constraining autophagy in renal tubules, promoting pyroptotic/apoptotic injury in SAKI. To interrogate this, we combined a CLP model with TREM-1 deficiency and pharmacologic NLRP3 modulation, alongside complementary tubular-cell gain-/loss-of-function studies.

## Methods

### Animals, Trem1 knockout generation, and cecal ligation and puncture (CLP) model

The Trem1 locus was edited by CRISPR/Cas9 on a C57BL/6J background. Based on the Trem1 transcript ENSMUST00000048782.6, exons 2–3 (coding region ~ 541 bp) were targeted to disrupt protein function. sgRNAs were transcribed in vitro and co-microinjected with Cas9 mRNA into fertilized C57BL/6J zygotes. Founders (F0) were identified by PCR and Sanger sequencing. F0 founders were crossed to C57BL/6J to establish F1 lines. Trem1-KO mice were generated by GemPharmatech (Nanjing, China).

Male C57BL/6J mice (12–15 weeks, 25 ± 5 g) were obtained from Charles River (Beijing, China). Mice were housed SPF at 22 ± 2 °C, 50 ± 5% humidity, 12-h light/dark, ad libitum chow/water, in accordance with NIH guidelines. Anesthesia and peri-operative analgesia were provided. Inject 0.3–0.5 ml of a 1.25% tribromoethanol solution (20 mg/ml, Aibei biotech) intraperitoneally per 10 g of mouse body weight. All animal experiments were conducted in strict accordance with the ARRIVE guidelines and the National Institutes of Health Guide for the Care and Use of Laboratory Animals. All procedures were approved by the Institutional Animal Care and Use Committee (IACUC) of Peking Union Medical College Hospital (protocol XHDW-2019-025).

Cecal ligation and puncture (CLP) was performed as described with the following parameters: the cecum was ligated at half the distance between the distal tip and the base (below the ileocecal valve), then two through-and-through punctures with a 21-gauge needle were made; a small droplet of feces was gently extruded from each puncture to ensure patency; the cecum was returned to the peritoneal cavity and the abdominal wall was closed (peritoneum/muscle and skin with 4 − 0 nylon)^18^. Mice received pre-warmed saline (1 mL, s.c.) for resuscitation and were maintained on a heating pad with no antibiotics. MCC950 (50 mg/kg)^19^ and nigericin (2.5 mg/kg)^20^ were administered intraperitoneally (i.p.) 12 h before CLP. The pre-treatment schedule was chosen a priori to ensure pathway modulation was established during the earliest phase of CLP-induced systemic inflammation, thereby enabling a mechanistic “proof-of-concept” test of NLRP3-dependent injury signaling under standardized exposure conditions. Mice were randomized by computer-generated simple randomization with concealed allocation (opaque sealed envelopes), stratified by genotype (WT vs. Trem1-KO) to ensure balanced group sizes. (1) WT-sham; (2) WT-CLP; (3) WT-CLP+MCC950 (50 mg/kg)^19^; (4) KO-sham; (5) KO-CLP; (6) KO-CLP+nigericin (2.5 mg/kg)^20^. The 24-h post-CLP time point was selected a priori as a reproducible acute-injury window in CLP sepsis models, during which renal dysfunction and tubular injury signals are robustly detectable, whereas later time points are more susceptible to survivor bias and phase heterogeneity. Accordingly, our mechanistic analyses were anchored to this acute phase snapshot, and time-course studies will be required to delineate initiation/progression/resolution dynamics. At 24 h post-CLP, blood and kidneys were collected under deep anesthesia for downstream assays (histology, cytokines, creatinine/BUN, and immunoblot). The mice (weighing 20–25 g) were euthanized under deep anesthesia (tribromoethanol 20 mg/mL, 0.3–0.5 mL per 10 g body weight, intraperitoneal). After loss of reflexes was confirmed, euthanasia was completed by cervical dislocation, consistent with AVMA Guidelines for the Euthanasia of Animals (2020).

A priori, we targeted *n* = 6 surviving mice per group at 24 h post-CLP for endpoint analyses. Because moderate-severity CLP is associated with substantial early mortality, we prospectively inflated the initial enrollment using an attrition-adjusted approach: *N* = 6 / (1-0.4expected mortality) per group to account for anticipated deaths.

Experiments were conducted in sequential cohorts, and additional animals were enrolled as needed until each septic group reached six 24-h survivors, according to this pre-specified stopping rule that was independent of outcome measurements. All sham/control animals (WT-sham and Trem1-KO-sham) survived to 24 h.

### Cell culture and cell treatment

HK-2 cells (ATCC) were cultured in DMEM/F-12 with 10% FBS, 100 U/mL penicillin, and 100 µg/mL streptomycin at 37 °C, 5% CO₂. Cells were plated at 3 × 10^5/well (6-well) and allowed to adhere for 8 h before transfection.

For the depletion of TREM-1, TREM-1 siRNA (Cat# sc-42999, Santa Cruz Biotechnology, USA), control shRNA plasmid (Cat# sc-108060, Santa Cruz Biotechnology, USA), TREM-1 cDNA ORF clone (Cat# HG10511-UT, Sino Bio, Shanghai, China), and negative control vector (Cat# CV011, Sino Bio, Shanghai, China) were transfected into HK-2 cells using Lipofectamine 3000 (Thermo Fisher Scientific, Waltham, Massachusetts)^16^. Cells were stimulated with lipopolysaccharide (LPS) from Escherichia coli O111:B4 (Sigma-Aldrich, L2630, phenol-extracted) at 100 ng/mL for the indicated times.

### Light microscopy specimen preparation and morphological observation

Kidney tissue was collected and fixed in 10% formalin solution immediately after euthanasia^16^. The tissue samples were dehydrated and embedded in paraffin wax. Serial paraffin Sect. (4 μm) were obtained and kept at 37 °C for more than 12 h. The sections were subjected to three consecutive washes with xylol for 5 min to remove paraffin and then hydrated by five consecutive washes with a descending alcohol content, 100, 95, 80, 70, and 50%, followed by deionized water. The histological paraffin sections were then stained with HE and Masson. Changes in the organizational structures were visualized using a light microscope. Two professional pathologists were asked to score the degree of kidney injury using a blinded method. Tubular injury was defined as tubular dilation, tubular atrophy, tubular cast formation, sloughing of tubular epithelial cells, or thickening of the tubular basement membrane. Only cortical tubules were included in the following scoring system: score 0, no tubular injury; score 1, less than 10% of tubules injured; score 2, 10 to 25% of tubules injured; score 3, 26 to 50% of tubules injured; score 4, 51 to 75% of tubules injured; and score 5, more than 75% of tubules injured. To ensure the accuracy of the score, three pathological sections were randomly selected from each animal, and 3 fields were randomly selected for review to obtain the average pathological score of kidney injury^21^.

### qRT‒PCR

Total mRNA from kidney tissue or TECs was extracted using the RNeasy^®^ Micro kit (Qiagen,, Hilden, Germany). The RNA quantity was measured with a Nanodrop ™ 2000 C spectrophotometer (Thermo Fisher Scientific, Waltham, Massachusetts) and reverse transcribed using iScript™ reverse transcription supermix for qRT‒PCR (Bio-Rad Laboratories, Inc., Hercules, CA, USA) according to the manufacturer’s instructions and our previous study^16^. The sequences of the TREM-1 forward and reverse primers were ATGAAGTATGCCAACAGCCAGAAGG and GGACAGGATGGAAGAGCACAACAG, respectively.

### Enzyme-linked immunosorbent assay (ELISA) and spectrophotometry

The concentrations of sTREM-1 (SEA213Hu&Mu), IL-1β (SEA563Hu&Mu), tumor necrosis factor α (TNFα) (SEA133Hu&Mu), and IL-6 (SEA079Hu&Mu) in the cell-free culture supernatants and serum were measured using commercially available specific ELISA kits (Wuhan USCN Business Co., Ltd., China). The Creatinine Assay Kit and Urea Assay Kit were used to detect the serum creatinine (SCR) and blood urea nitrogen (BUN) levels by spectrophotometry (C011-1-1, C013-1-1 Nanjing Jiancheng Co., Ltd., China). ELISA and spectrophotometry were performed in duplicate, and the other assays were performed in strict accordance with the manufacturer’s instructions.

### Western blotting analysis

Western blotting analysis was performed using standard procedures. TREM-1 antibody was purchased from Absin (abs135611, 1:500). Cleaved caspase-3 (Asp175) antibody (#9661, 1:1000), cleaved caspase-1 (Asp296) (E2G2I) rabbit mAb (#89332, 1:1000), Bcl-2 (D17C4) rabbit mAb (#3498, 1:1000), Bax antibody (#2772, 1:1000), NLRP3 (D4D8T) rabbit mAb (#15101, 1:1000), cleaved gasdermin D (Asp276) (E3E3P) rabbit mAb (#10137, 1:1000), Atg5 (D5F5U) rabbit mAb (#12994, 1:1000), GAPDH (#5174, 1:2000), and β-actin (#4967, 1:2000) were obtained from Cell Signal Technology. LC3b (#Proteintech, 14600-1-AP, 1:1000) was obtained from ProteinTech. An antibody for beclin-1 (purified mouse anti-beclin, BD# 612113) was obtained from BD. Horseradish peroxidase-conjugated secondary antibodies were used, and specific antibody-antigen complexes were detected using a chemiluminescent substrate.

### Statistical analysis

The experiments were performed in at least triplicate, and the data are expressed as the mean ± standard deviation (SD). One-way ANOVA was used for comparisons among the different groups. The results for continuous variables that were not normally distributed were compared using nonparametric tests. P values < 0.05 were considered to indicate statistical significance. The data were analyzed using SPSS 13.0 software (SPSS Inc. in Chicago, IL, USA).

## Results

### CLP induces renal TREM-1 and knockout abolishes expression

**Fig. 1:**
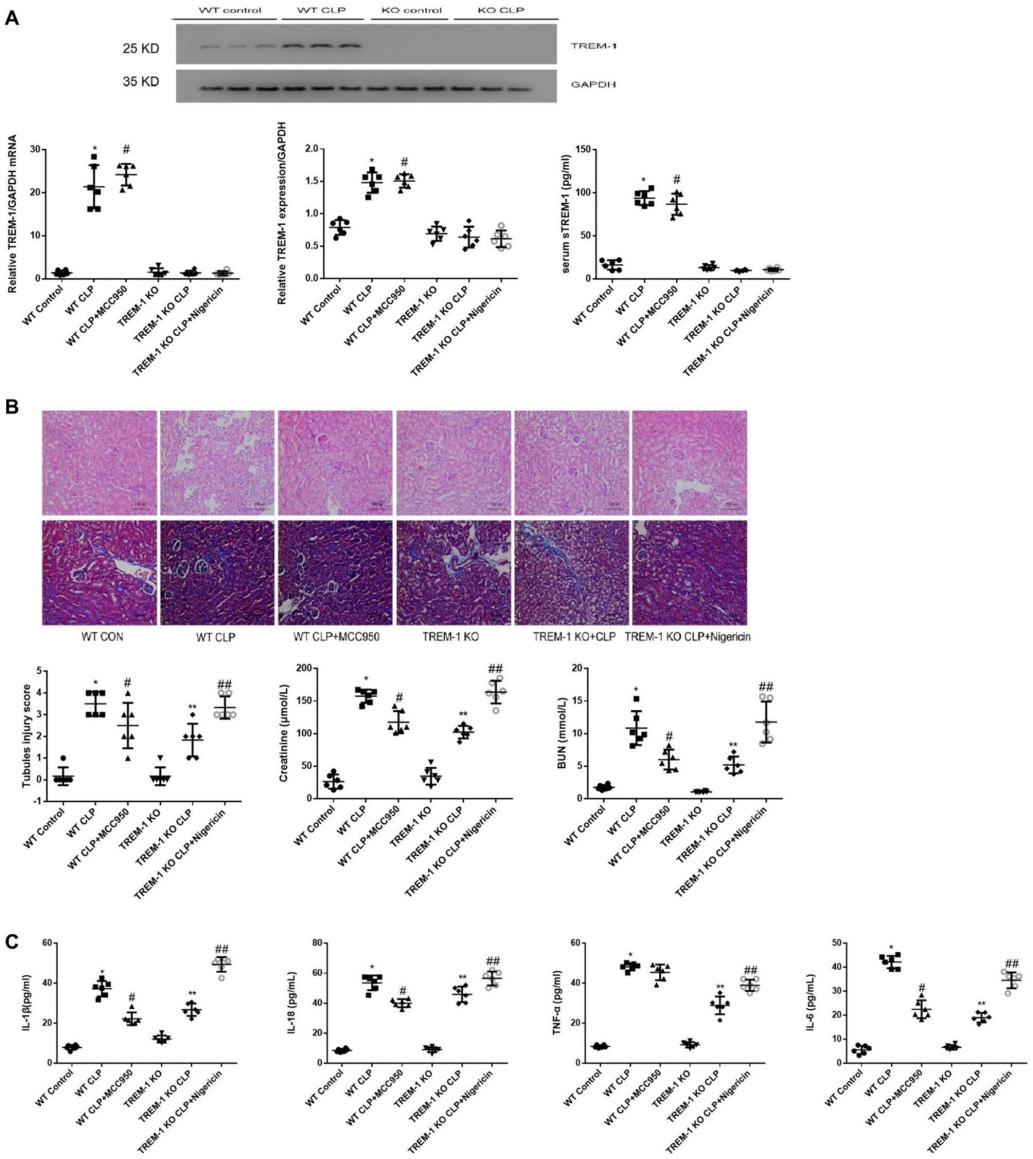
AKI model, established by CLP, in TREM-1-KO and WT control mice. The effects of NLRP3 deletion (MCC950) and activation (nigericin) on the NLRP3 inflammasome in CLP-AKI mice were also examined. *Panel A*: Kidney TREM-1 protein and mRNA expression and serum sTREM-1 concentration in the different groups determined by Western blotting, PCR and ELISA (*n* = 6 per group). *Panel B*: Effect of renal injury after CLP on renal morphology. Representative micrographs showing kidney injury in the different groups of mice (original magnification is ×200). The kidney sections were subjected to HE and Masson staining. The kidney injury scores were quantitatively determined. The effect of renal injury on serum creatine and BUN levels was assessed (*n* = 6 per group). *Panel C*: TREM-1 mediates the production of inflammatory cytokines (IL-1β, TNF-α, and IL-6) in the different groups (*n* = 6 per group). The data are presented as the means ± SDs. One-way ANOVA (normal distributions) or nonparametric tests (not normal distributions) were used for comparisons among the different groups. * indicates *P* < 0.05 vs. the WT control group; # indicates *P* < 0.05 vs. the WT + CLP group; ** indicates *P* < 0.05 vs. the TREM-1 KO group; and ## indicates *P* < 0.05 vs. the TREM-1 KO + CLP group.

We generated a Trem1 knockout (Trem1-KO) mouse line. At 24 h after CLP, renal Trem1 mRNA and protein were increased in WT mice but absent in Trem1-KO (Fig. 1A). WT kidneys showed higher Trem1 expression versus sham, whereas Trem1-KO showed no detectable expression.Serum sTREM-1 likewise increased after CLP in WT but not in Trem1-KO. TREM-1 expression in the WT mice was significantly higher after CLP (93.88 ± 8.19 pg/mL) than that in the WT + control mice (22.10 ± 16.33 pg/mL).

Histology demonstrated more severe tubular injury in WT-CLP versus WT-sham (Fig. 1B). Findings included tubular dilation/atrophy, casts, epithelial sloughing, and brush-border loss, prominent at the corticomedullary junction. Tubular-injury score: 3.50 ± 0.55 vs. 0.17 ± 0.41 (*P* < 0.001; *n* = 6). BUN 10.86 ± 2.61 vs. 1.76 ± 0.37 mmol/L; creatinine 157.52 ± 10.16 vs. 26.50 ± 10.91 µmol/L (both *P* < 0.001). Systemic cytokines increased after CLP (Fig. 1C). WT-CLP vs. WT-sham: IL-1β 37.28 ± 3.85 vs. 7.94 ± 1.18 pg/mL; TNF-α 48.21 ± 1.81 vs. 8.40 ± 0.69 pg/mL; IL-6 42.18 ± 2.56 vs. 5.59 ± 1.71 pg/mL (all *P* < 0.05; *n* = 6).

### Trem1 deficiency attenuates CLP-induced injury while NLRP3 modulation aligns with the axis

Trem1-KO reduced tubular-injury score (1.83 ± 0.75 vs. 3.50 ± 0.55; *P* < 0.05), BUN (5.20 ± 1.29 vs. 10.86 ± 2.61 mmol/L), and creatinine (102.48 ± 9.67 vs. 157.52 ± 10.16 µmol/L) compared with WT-CLP (Fig. 1B). Serum IL-1β, TNF-α, and IL-6 were lower in Trem1-KO-CLP than WT-CLP (Fig. 1C).: In vivo NLRP3 modulation recapitulated the axis: MCC950 reduced injury in WT-CLP without altering Trem1 levels, whereas nigericin aggravated injury in Trem1-KO-CLP (Fig. 1B–C).Across groups, higher Trem1/sTREM-1 associated with greater injury and cytokine levels (trend consistent with Fig. 1).

### Trem1 deficiency suppresses NLRP3 inflammasome/pyroptosis signaling with parallel reductions in apoptosis markers

**Fig. 2:**
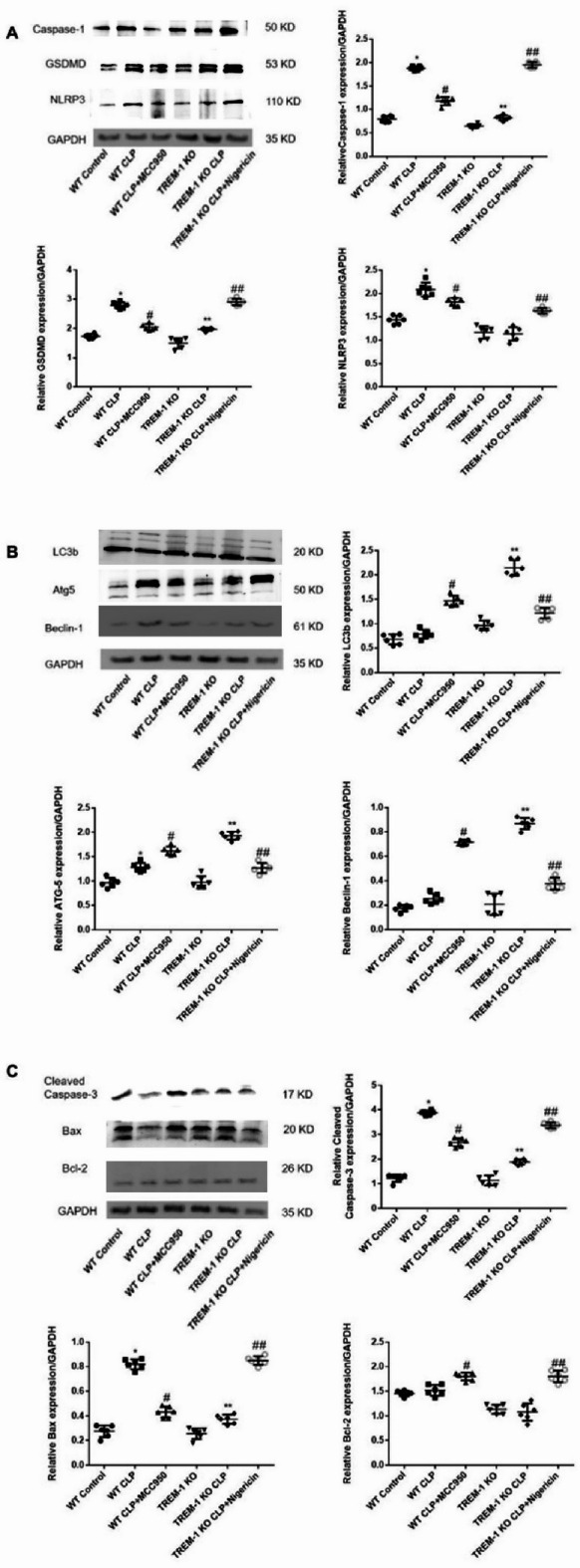
Effect of TREM-1 on inflammasome activation, autophagy and apoptosis changes in the kidneys of CLP-AKI mice (*n* = 6). *Panels A-C*: Representative Western blots and quantitative determination of the levels of proteins related to inflammasome activation, autophagy and apoptosis are presented. The data are presented as the means ± SDs. One-way ANOVA (normal distributions) or nonparametric tests (not normal distributions) were used for comparisons among the different groups. * indicates *P* < 0.05 vs. the WT control group; # indicates *P* < 0.05 vs. the WT + CLP group; ** indicates *P* < 0.05 vs. the TREM-1 KO group; and ## indicates *P* < 0.05 vs. the TREM-1 KO + CLP group.

Versus WT-CLP, Trem1-KO-CLP showed lower NLRP3, cleaved caspase-1, and cleaved GSDMD (Fig. 2A). Autophagy markers (Beclin-1, Atg5, LC3B) were increased in TREM1-KO-CLP.(Fig. 2B) Tharmacologic NLRP3 inhibition (MCC950) further increased autophagy markers, whereas NLRP3 activation (nigericin) decreased them, indicating an inverse association between NLRP3 activity and autophagy. Apoptosis markers, indluding cleaved caspase-3 and Bax, were reduced in TREM1-KO-CLP and higher in WT-CLP (Fig. 2C).

### TREM-1 gain/loss in HK-2 and LPS responsiveness

**Fig. 3:**
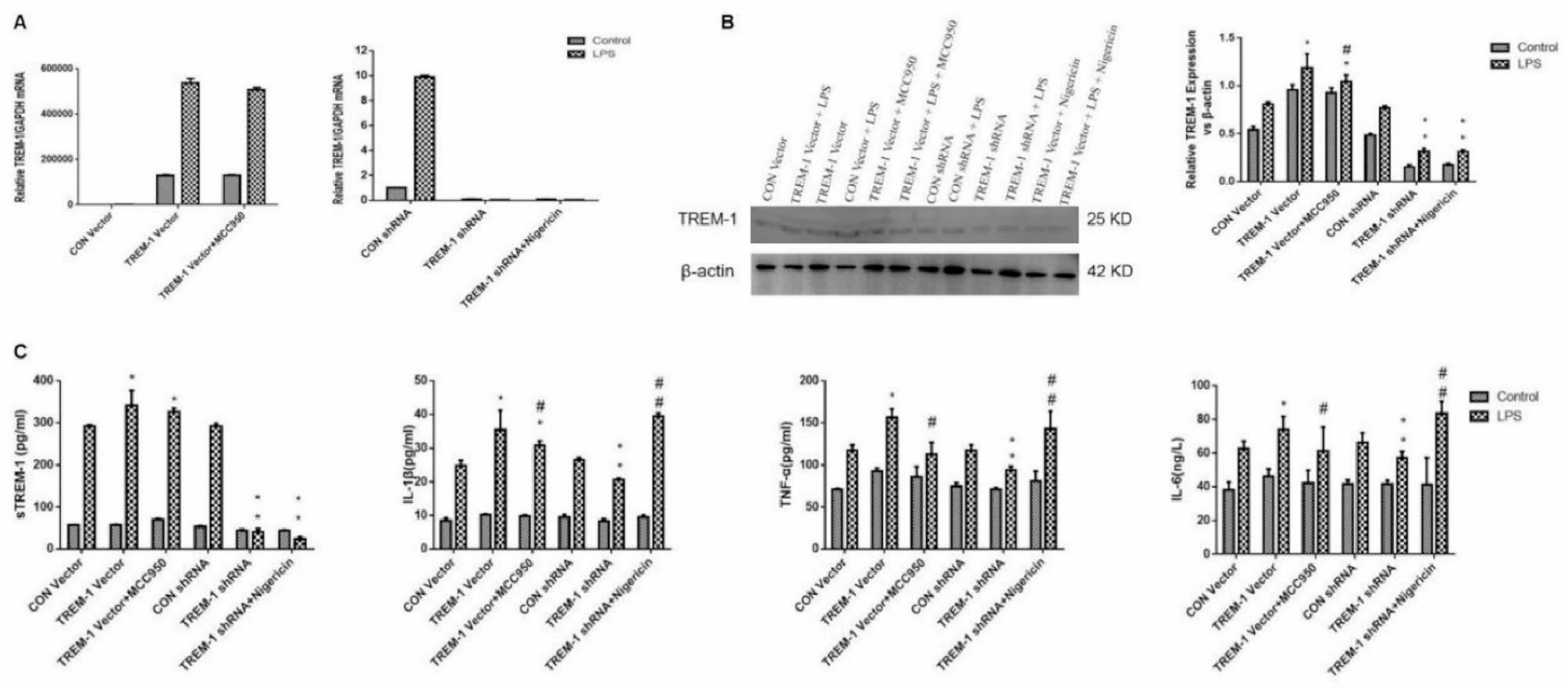
Activation or silencing of TREM-1 expression in HK-2 cells by transfection of TREM-1 vector or shRNA and treatment with or without LPS. HK-2 cells were transfected with a plasmid containing the negative control vector/TREM-1 gene or negative control shRNA/TREM-1 shRNA and then exposed to LPS (100 ng/mL). Additionally, an NLRP3 inhibitor (MCC950) and an NLRP3 activator (nigericin) were administered to HK-2 cells. *Panel A*: Representative qRT-PCR and quantification of TREM-1 in the different groups. *Panel B*: Western blotting analysis and quantification of TREM-1 in the different groups. *Panel C*: Inflammation and cytokine expression, including sTREM-1, IL-1β, TNFα, and IL-6, in HK-2 cell supernatants. Three samples were included per group. The data are presented as the means ± SDs. One-way ANOVA (normal distributions) or nonparametric tests (not normal distributions) were used for comparisons among the different groups. * indicates *P* < 0.05 vs. the CON vector group; # indicates *P* < 0.05 vs. the TREM-1 vector group; ** indicates *P* < 0.05 vs. the CON shRNA group; ## indicates *P* < 0.05 vs. the TREM-1 shRNA group; and ^ indicates *P* < 0.05 between the TREM-1 vector and TREM-1 shRNA groups.

RT-qPCR and Western blot confirmed successful TREM-1 overexpression or knockdown in HK-2 (Fig. 3A, B). Both manipulations were effective.LPS further increased TREM-1 in vector-transfected cells; TREM-1 overexpression augmented, while TREM-1 shRNA attenuated, this induction. In supernatants, sTREM-1 increased with LPS; TREM-1 overexpression raised, whereas TREM-1 knockdown lowered sTREM-1 versus respective controls (Fig. 3C). IL-1β, TNF-α, and IL-6 showed concordant changes.

### TREM-1 regulates the NLRP3–autophagy balance and apoptosis in HK-2

**Fig. 4:**
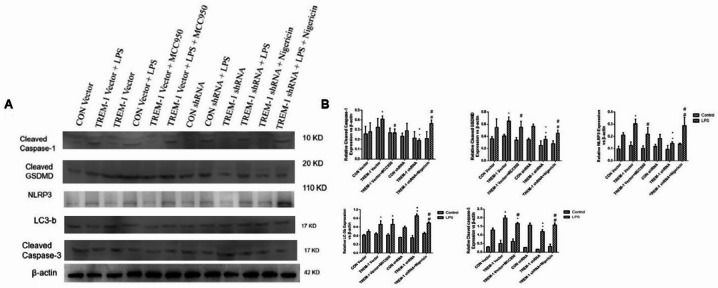
LPS-induced HK-2 cell inflammasome, autophagy, and apoptosis mediated by TREM-1. HK-2 cells were transfected with a plasmid containing the negative control vector/TREM-1 gene or negative control shRNA/TREM-1 shRNA and then exposed to LPS (10 µg/mL). Additionally, an NLRP3 inhibitor (MCC950) or an NLRP3 activator (nigericin) were administered to HK-2 cells. Representative Western blots (panel A)and quantitative determination of protein levels (panel B) are presented. Three samples were included per group. The data are presented as the means ± SDs. One-way ANOVA (normal distributions) or nonparametric tests (not normal distributions) were used for comparisons among the different groups. * indicates *P* < 0.05 vs. the CON vector group; # indicates *P* < 0.05 vs. the TREM-1 vector group; ** indicates *P* < 0.05 vs. the CON shRNA group; and ## indicates *P* < 0.05 vs. the TREM-1 shRNA group.

Upon LPS, inflammasome markers increased. Autophagy readouts were modulated in opposite directions by TREM-1 gain/loss, consistent with group-specific responses (Fig. 4). Densitometry showed that TREM-1 overexpression increased cleaved caspase-1 and cleaved GSDMD and reduced LC3B, while TREM-1 knockdown produced the opposite pattern. Relative to unstimulated controls, LPS increased inflammasome markers and lowered autophagy markers. Collectively, TREM-1 overexpression was associated with higher inflammasome activity and apoptosis and lower autophagy, whereas TREM-1 inhibition showed the reciprocal pattern; MCC950 and nigericin produced mirror pharmacologic effects.

## Discussion

**Fig. 5:**
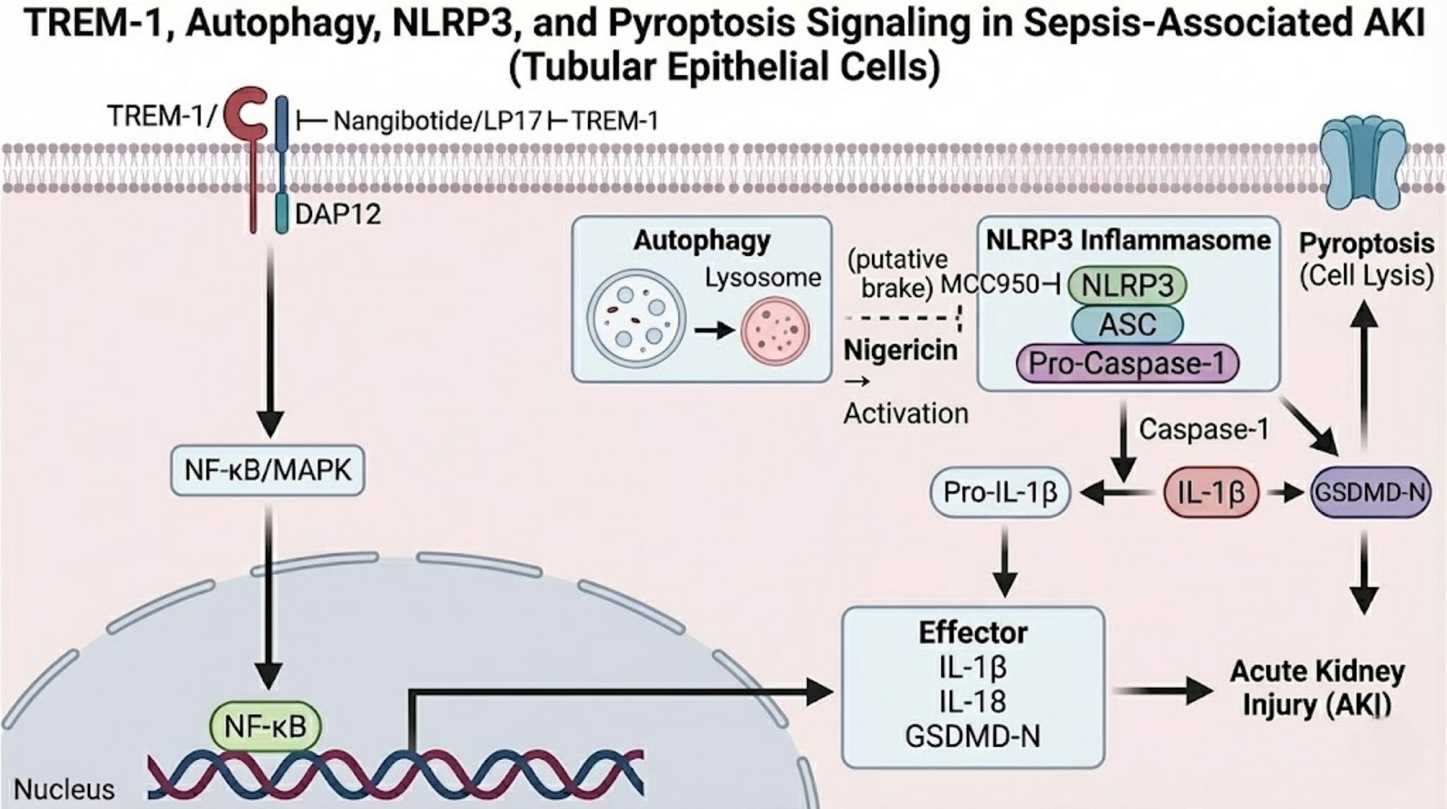
Proposed TREM-1–autophagy–NLRP3–pyroptosis signaling axis in sepsis-associated AKI. Schematic illustration of the working model derived from this study. In tubular epithelial cells during sepsis, activation of TREM-1 signals through the adaptor DAP12 to engage NF-κB/MAPK pathways, providing a priming signal that upregulates inflammasome components and pro-IL-1β. Subsequent activation of the NLRP3 inflammasome leads to caspase-1 activation, cleavage of pro-IL-1β and gasdermin D (GSDMD), and execution of pyroptotic cell death, culminating in tubular injury and acute kidney injury (AKI). Autophagy is depicted as a putative counter-regulatory “brake” on inflammasome activation, consistent with the observed inverse association between autophagy markers and NLRP3–pyroptosis readouts in this study. Pharmacologic modulation is indicated: MCC950 inhibits NLRP3 activation, whereas nigericin provides an activation signal for the inflammasome. TREM-1 inhibition (e.g., nangibotide or LP17) attenuates upstream inflammatory amplification.

Using a CLP model with *TREM-1*-KO mouse model, we observed markedly reduced tubular injury and systemic inflammation, accompanied by blunted renal NLRP3 inflammasome activation and pyroptosis In tubular epithelial cells, TREM-1 overexpression increased NLRP3 activation, IL-1β release, and pyroptosis markers, whereas TREM-1 knockdown produced the opposite pattern. In vivo, pharmacologic modulation of NLRP3 aligned with this axis, MCC950 attenuated kidney injury in WT-CLP mice, while nigericin aggravated injury in *TREM-1*-KO mice, supporting a TREM-1-NLRP3 pyroptosis pathway as a principal driver of S-AKI in our model. Additionally, TREM-1 deficiency was associated with higher autophagy markers (e.g., LC3B, Beclin-1, Atg5). The potential mechanisms can be referred to Fig. 5.

Triggering receptor expressed on myeloid cells-1 (TREM-1) is a DAP12-coupled immunoreceptor predominantly on neutrophils and monocytes/macrophages that amplifies innate inflammatory signaling upon ligand engagement. TTREM-1 cooperates with Toll-like and NOD-like receptor pathways to potentiate downstream NF-κB/MAPK cascades during septic inflammation^22^. Its soluble form (sTREM-1), generated by shedding, increases in circulation during infection and tracks inflammatory burden. Our clinical work showed that serum sTREM-1 aids early sepsis diagnosis and can outperform or complement PCT and CRP^23^. The dynamics of sTREM-1 in serum and its gene polymorphisms are related to the prognosis of sepsis patients^24^. Higher sTREM-1 levels correlate with illness severity in ICU sepsis cohorts, consistent with other inflammatory markers^23,25^. A meta-analysis from our group further linked higher sTREM-1 concentrations to increased mortality risk in infected adults^23,26^, consistent with proposed mechanistic links^11^. Experimentally, LPS upregulated TREM-1 in renal tubular epithelium, accompanied by reduced autophagy readouts and aggravated injury phenotypes in vitro^16^. Pharmacologic blockade of TREM-1 mitigates systemic inflammation and improves survival in polymicrobial sepsis models^27^. Consistent with these observations, our Trem1-deficient septic mice and TREM-1–silenced tubular cells exhibited attenuated inflammatory responses and injury. Notably, TREM-1 may exert context-dependent effects during repair; deficiency can impair recovery after sterile ischemic injury in some settings^28^. Such phase- and context-specific effects likely depend on the primary insult, cell type, and inflammatory milieu; in fulminant septic AKI, our data support TREM-1 as an upstream amplifier of the NLRP3 ‘accelerator’ pathway.

TREM-1 signals through multiple downstream pathways; relevant to our findings, it lowers the threshold for inflammasome activation during septic inflammation. Among pattern-recognition pathways, the NLRP3 inflammasome appears central to the propagation of septic tissue injury. Upon sensing pathogen- or damage-associated cues, inflammasome complexes assemble and activate caspase-1^29^. Caspase-1 then matures IL-1β and IL-18 and cleaves gasdermin D, driving pro-inflammatory pyroptotic cell death. NLRP3 is a broadly responsive sensor that integrates diverse cellular stress signals rather than binding a single ligand. It is expressed in myeloid and parenchymal cells and requires both a priming signal (e.g., NF-κB–dependent transcription) and an activation signal, e.g., K⁺ efflux, ROS, mitochondrial stress, to engage^30^. Consistently, blocking TREM-1 attenuated LPS-induced lung injury in association with reduced NLRP3 activation^31^. DAMP-driven signals are particularly relevant to organ damage in severe infection. In experimental subarachnoid hemorrhage, TREM-1 exacerbated neuroinflammation via NLRP3-dependent inflammatory cell death^32^. The TREM-1 inhibitor LP17 likewise reduced ischemic neuronal injury by limiting oxidative stress and pyroptosis^33^. Extending these observations, our data place TREM-1 upstream of NLRP3 in both septic kidneys and tubular epithelial cells. Loss of TREM-1 limited NLRP3 activation and downstream caspase-1/GSDMD processing. Conversely, TREM-1 activation increased NLRP3 readouts and pyroptosis markers with concordant rises in IL-1β in WT mice and tubular cells. In renal ischemia–reperfusion, pyroptosis-related proteins (e.g., caspase-1, caspase-11, IL-1 family) are increased alongside structural and functional injury^34^. Genetic disruption of NLRP3 or ASC reduces inflammation, renal injury indices, and mortality in murine AKI models^35^. In unilateral ureteral obstruction, NLRP3 activation promotes IL-1β expression within kidney parenchyma, underscoring its role beyond immune cells^36^. Nevertheless, NLRP3-driven pyroptosis in renal tubular epithelium remains less well characterized in AKI. Our results support a DAMP-sensitive TREM-1-NLRP3 axis as a key accelerator of injury in septic AKI. Positioning TREM-1 upstream of NLRP3 in tubular cells highlights a tractable target for mitigating inflammasome-mediated kidney damage.

Experimental models indicate that autophagy is activated as a renoprotective response during endotoxemic AKI^37^. Clinically, higher expression of autophagy-related genes in AKI patients on CRRT has been associated with better outcomes^38^. Mechanistically, autophagy helps counter oxidative stress and removes damaged organelles/proteins in AK^14^. Loss of mitophagy regulators (Pink1/Parkin) aggravated injury and apoptosis in septic AKI models, supporting a protective role for mitochondrial autophagy^39^. Upstream modulators such as SIRT3 confer protection in CLP-induced AKI partly by enhancing autophagy via AMPK/mTOR signaling^40^. Dexmedetomidine increased autophagy through α2-AR/AMPK/mTOR, concomitantly limiting NLRP3 activation and reducing LPS-induced AKI^41^. Consistently, autophagy limits endotoxemic AKI and modulates tubular cytokine responses^15^. In our study, Trem1 deficiency was associated with higher renal autophagy markers (LC3B, Beclin-1, Atg5) and lower inflammatory readouts. We observed an inverse association between autophagy readouts and inflammasome activation. Reduced Trem1 activity coincided with lower inflammasome readouts; whether this is mediated by enhanced autophagy remains to be determined. Pharmacologic NLRP3 modulation showed directionally opposite changes in autophagy markers in vivo, consistent with a reciprocal relationship between these pathways. Parkin-mediated mitophagy has been shown to suppress NLRP3 activation and protect against septic AKI^42^. Inhibiting NLRP3 attenuated apoptosis and injury in contrast-induced AKI^43^. Mitochondrial quality control that augments autophagy was associated with reduced apoptosis in AKI and interacted with NLRP3 signaling^44^. Collectively, our data support a Trem1-NLRP3 induced pyroptosis ‘accelerator’ pathway as a principal driver of septic AKI, with autophagy changes observed in parallel. We hypothesize that autophagy may reflect a counter-regulatory ‘brake’ on NLRP3 activation, a testable premise for future rescue experiments. This interpretation is consistent with prior AKI literature indicating that tubular autophagy and mitophagy can restrain inflammasome signaling, while direct causality requires targeted autophagy perturbation^45^.

Our mechanistic data support a TREM-1–NLRP3–caspase-1–GSDMD axis as an inflammatory effector pathway in SA-AKI. In this context, soluble TREM-1 (sTREM-1) may be interpreted as a host-response readout of TREM-1 pathway activation (i.e., receptor shedding), which is conceptually complementary to established “tubular injury” biomarkers such as NGAL and KIM-1. Whereas NGAL and KIM-1 primarily reflect tubular stress/epithelial injury and have demonstrated diagnostic performance in SA-AKI^46^, sTREM-1 may add pathway specificity by reporting activation of an upstream immune-amplification circuit. Clinically, urinary (and in some studies plasma) sTREM-1 has shown diagnostic and/or predictive value for sepsis-associated AKI, including our prospective ICU cohort^8^ and an independent study directly comparing NGAL, cystatin C, and sTREM-1 in septic patients^9^. Together, these observations support a rationale for multi-marker strategies in which sTREM-1 complements injury markers rather than replacing them. However, a key translational gap remains: we did not correlate circulating/urinary sTREM-1 with renal inflammasome activation or pyroptosis signatures. Future prospective cohorts with serial plasma/urine sTREM-1 and concurrent assessment of AKI severity (KDIGO) should incorporate pathway-aligned surrogates (e.g., IL-18 and exploratory assays for gasdermin fragments in urine/extracellular vesicles) to directly link biomarker kinetics to the TREM-1–NLRP3 axis and to identify clinically actionable windows for TREM-1–targeted intervention.

Beyond biomarker utility, several strategies to inhibit or modulate TREM-1 signaling have been developed. The most clinically advanced agent is nangibotide (LR12), a peptide TREM-1 inhibitor/modulator. In a randomized phase 2a trial in septic shock, nangibotide demonstrated acceptable safety and pharmacokinetics, with pharmacodynamic signals consistent with attenuation of excessive inflammation^47^. In the multicenter phase 2b ASTONISH trial, nangibotide again showed an acceptable safety profile, although the primary endpoint was not met in the overall population; importantly, biomarker-enrichment analyses suggested greater organ dysfunction improvement among patients with higher baseline sTREM-1, supporting a precision strategy in which benefit may be concentrated in patients with demonstrable TREM-1 pathway activation^12^. In parallel, multiple preclinical TREM-1 inhibitory modalities—most notably the inhibitory peptide LP17—have shown reduced cytokine storm and improved outcomes in experimental sepsis models, supporting the biological plausibility of TREM-1 blockade^48^. Together with our mechanistic data linking TREM-1 to NLRP3-mediated pyroptosis in tubular epithelial cells, these findings motivate future biomarker-guided interventional studies that incorporate kidney-relevant endpoints in sepsis.

This study has several limitations. First, the observed increase in autophagy markers in parallel with reduced NLRP3 inflammasome–pyroptosis activity in Trem1-deficient settings should be interpreted as concomitant rather than causal, because we did not directly manipulate autophagy. In addition, autophagy was assessed mainly using static markers, which may reflect either increased induction or impaired turnover; therefore, definitive mechanistic attribution will require targeted autophagy perturbation together with standardized flux assays. Second, all in vivo endpoints were assessed at a single time point (24 h post-CLP), providing a standardized acute-injury snapshot but precluding phase-specific inference (initiation vs. propagation vs. resolution). Given the substantial early mortality and increasing phase heterogeneity in moderate-to-severe CLP, which can introduce survivor/attrition bias at later time points, we anchored mechanistic analyses to 24 h and ensured a pre-specified number of 24-h survivors per group. Future work should incorporate multi-time-point profiling (e.g., 6–12 h and 48–72 h) and post-insult intervention to define actionable therapeutic windows. Thirdly, all experiments were performed in male mice, which may limit generalizability. Given reported sex differences in immune regulation, inflammasome signaling, and susceptibility to AKI, future studies should include both sexes to determine whether the TREM-1–NLRP3–pyroptosis axis exhibits sex-dependent effects. Finally, the translational bridge remains incomplete. We did not correlate renal Trem1/NLRP3 activity with circulating or urinary biomarkers; prospective cohorts linking plasma/urine sTREM-1 to renal inflammasome/pyroptosis signatures (e.g., IL-18, GSDMD fragments) and clinically relevant renal outcomes, as well as interventional studies that include kidney endpoints, will be critical to strengthen clinical applicability. Together, these steps will refine the mechanistic framework and improve the translational relevance of targeting the TREM-1–NLRP3 “accelerator” axis in sepsis-associated AKI.

## Conclusion

In summary, our data position TREM-1 as an upstream amplifier of the NLRP3 inflammasome and pyroptotic injury in sepsis-associated AKI (S-AKI). Genetic Trem1 loss in vivo and TREM-1 modulation in tubular cells consistently shifted readouts toward lower NLRP3/caspase-1/GSDMD activity, reduced cytokine release, and attenuated tubular damage.Autophagy marker changes accompanied Trem1 deficiency but were not manipulated; thus, we interpret them as concomitant and present a “gas-and-brake” framework as a testable hypothesis, in which TREM-1 robustly amplifies NLRP3-driven pyroptosis while autophagy may reflect a parallel, potentially counter-regulatory process that requires functional validation. Collectively, these findings support sTREM-1 as an early biomarker and nominate TREM-1 as a therapeutic target, motivating prospective biomarker–outcome studies and evaluation of post-insult TREM-1 blockade in S-AKI.

### Statement of Ethics

This study did not involve human participants. All animal procedures complied with institutional guidelines and were approved by the PUMCH IACUC (XHDW-2019-025).

## Supplementary Information

Below is the link to the electronic supplementary material.

Supplementary Material 1

## Data Availability

Any datasets used can be accessed by contacting the corresponding author.
