# Supplementary material for: TREM1–NLRP3–driven pyroptosis in sepsis-associated acute kidney injury (AKI) with parallel autophagy changes
Source: Sci Rep. 2026 Mar 19;16:14239. doi: 10.1038/s41598-026-40893-w (PMC13139595; doi:10.1038/s41598-026-40893-w)

FIGURE 1A

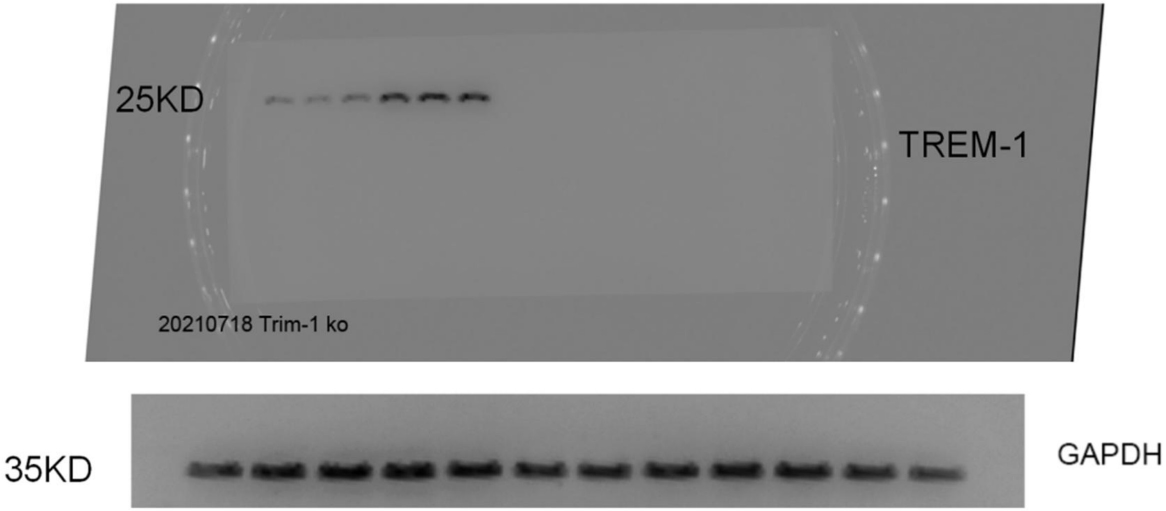

FIGURE 2A

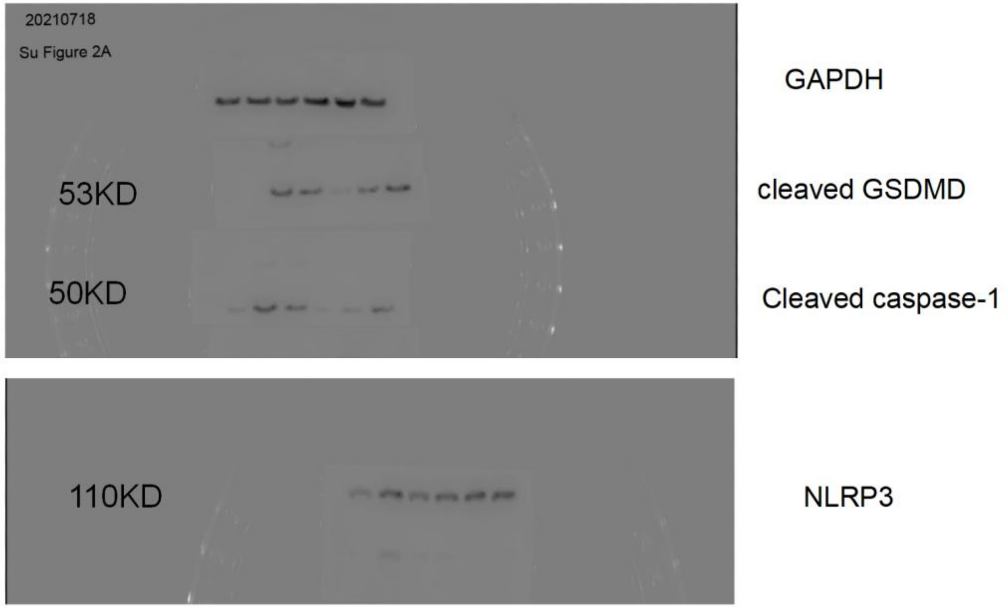
20210718
Su Figure 2A
GAPDH
53KD
cleaved GSDMD
50KD
Cleaved caspase-1
110KD
NLRP3

Figure2A

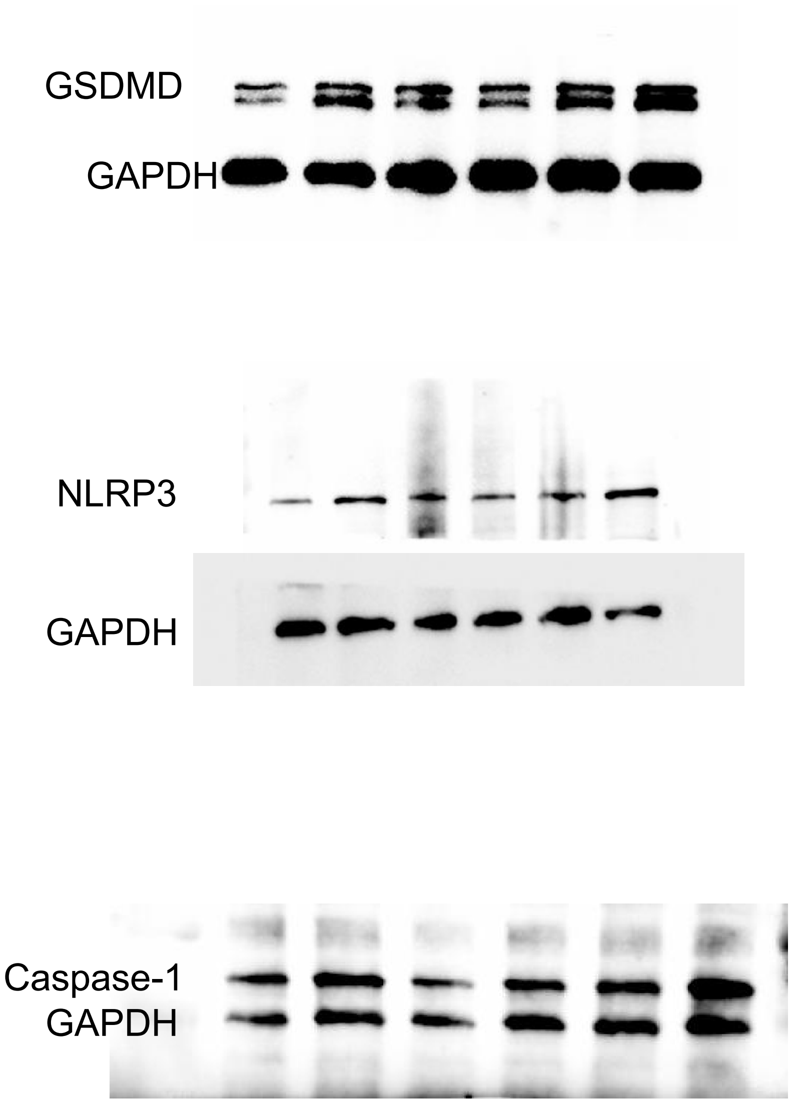

FIGURE 2B

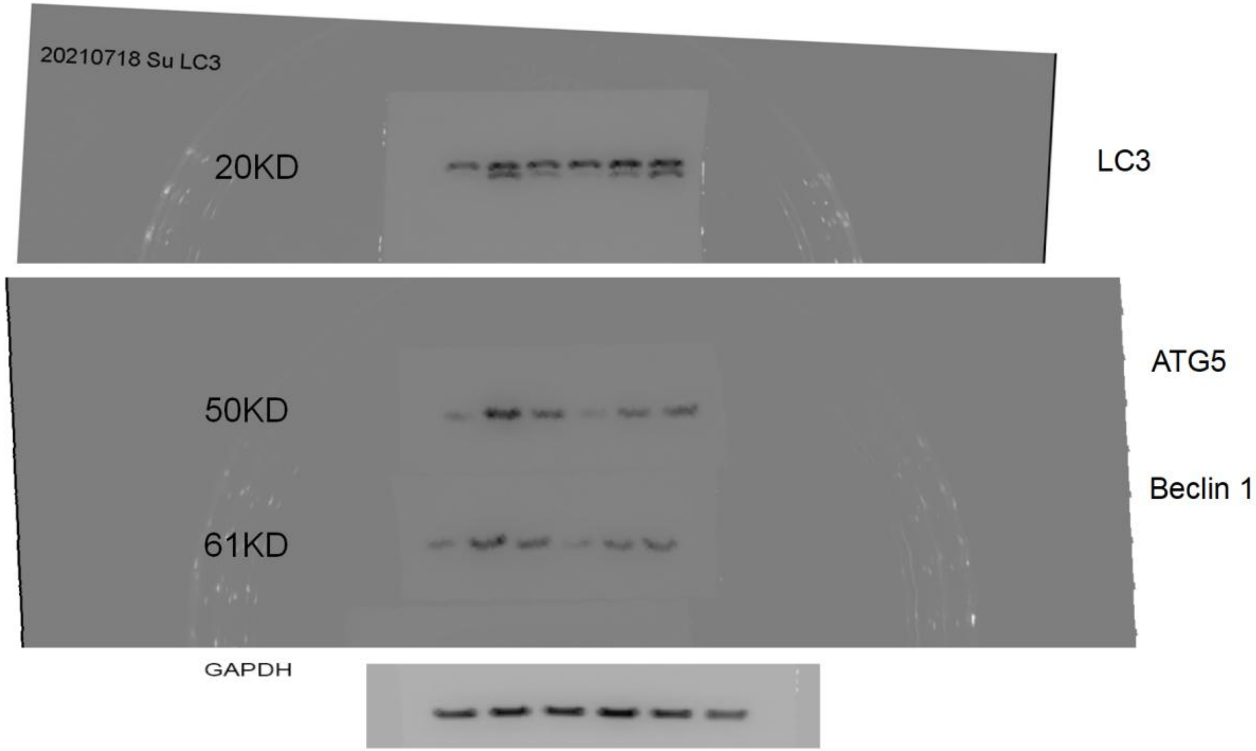

Figure2B

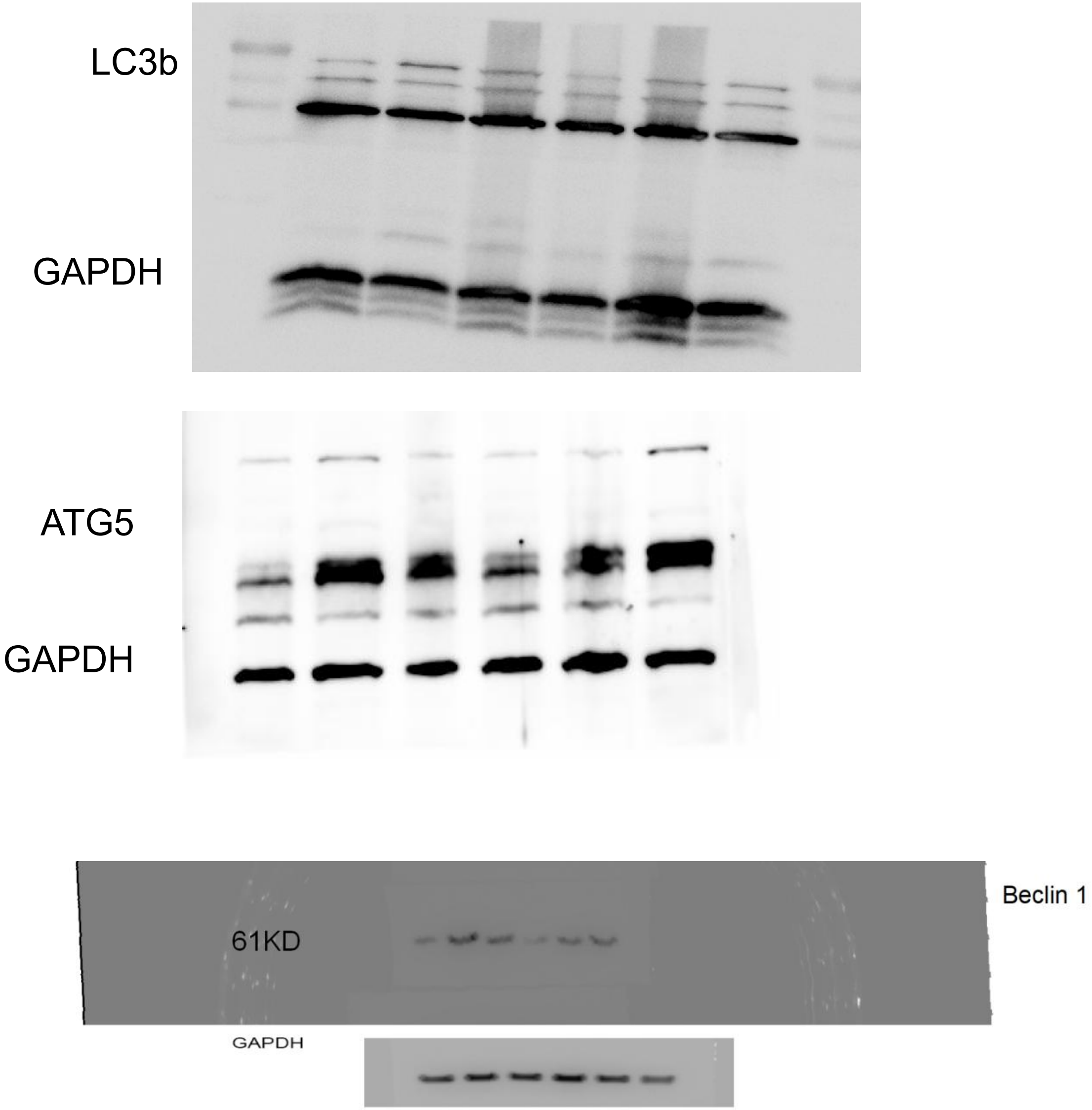

FIGURE 2C

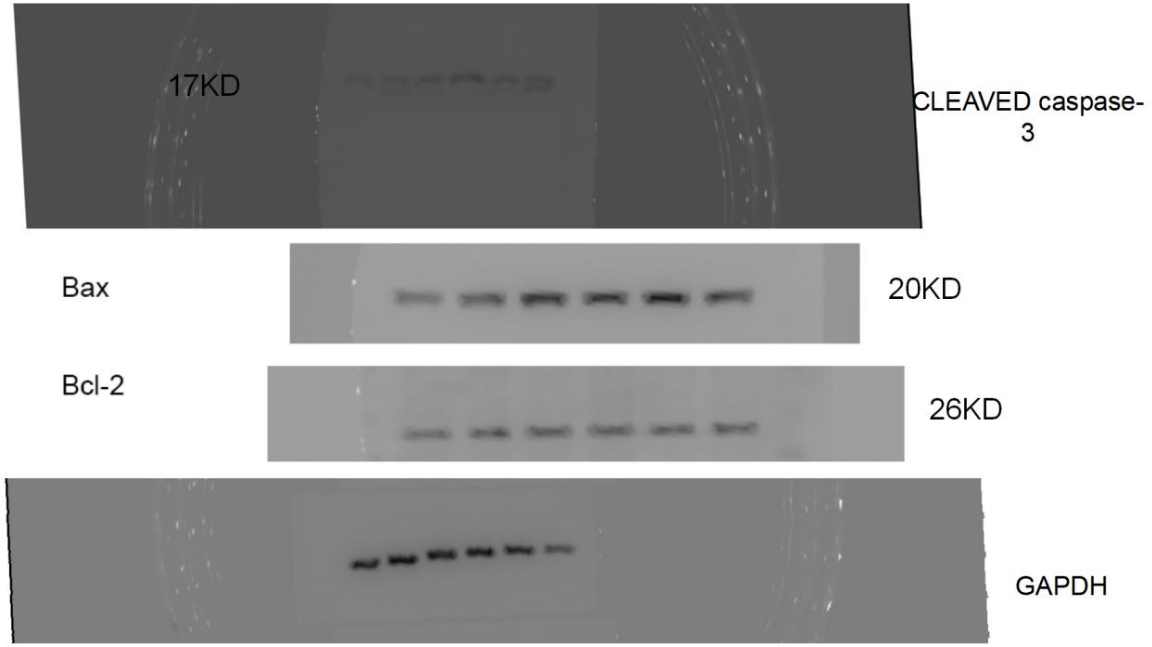

Figure2C

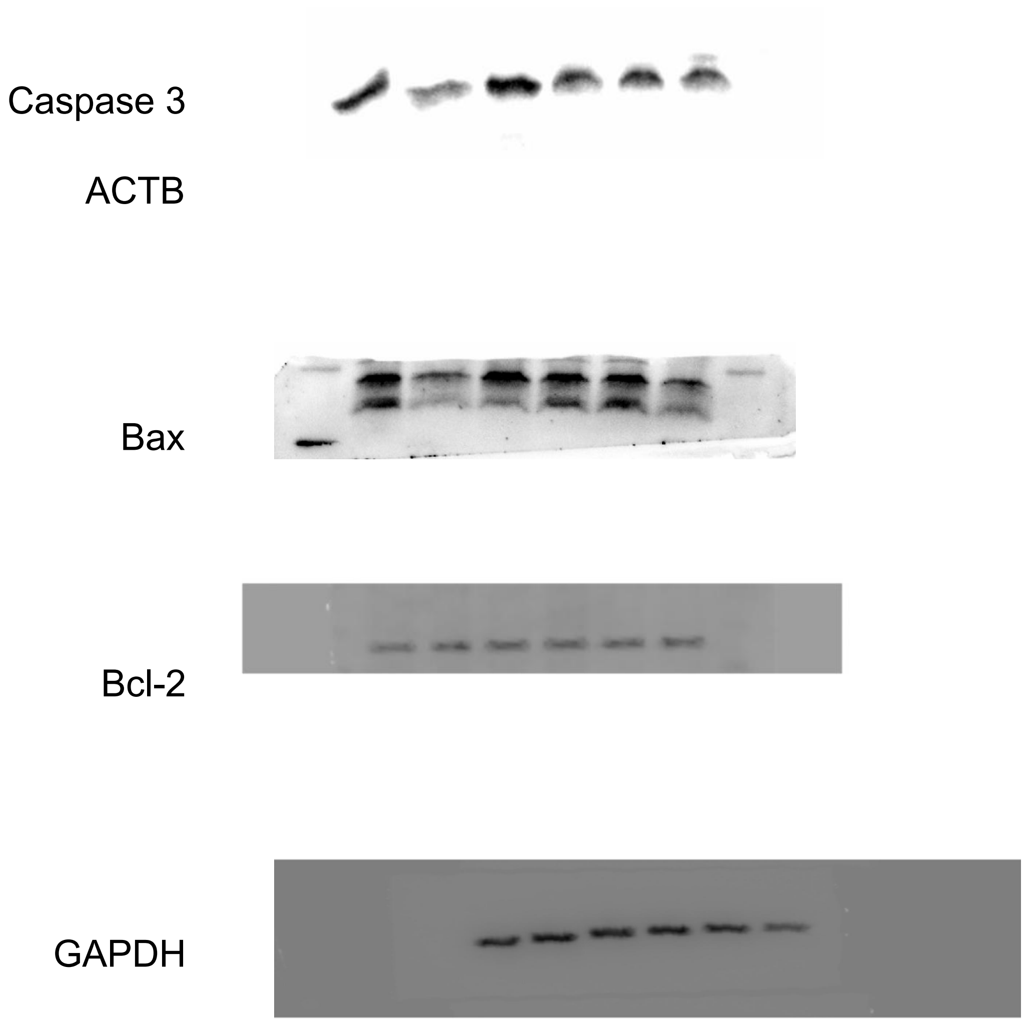

FIGURE 3B

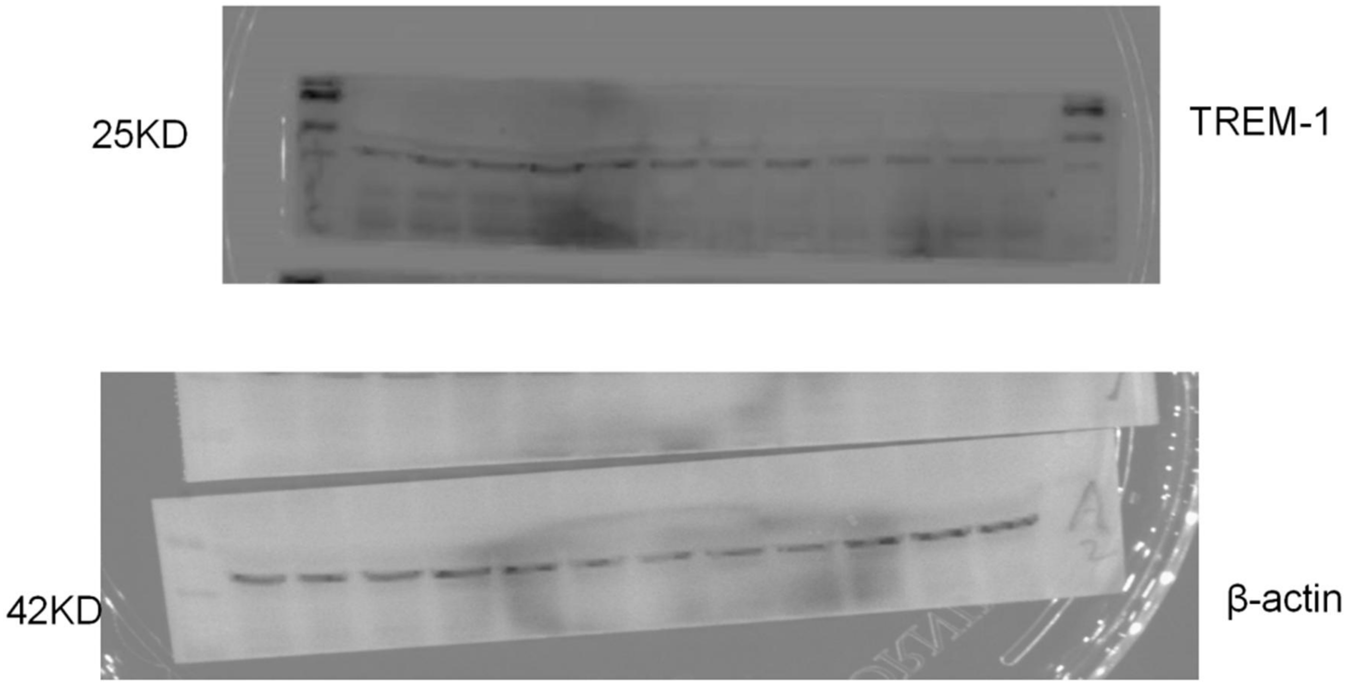

FIGURE4A-1

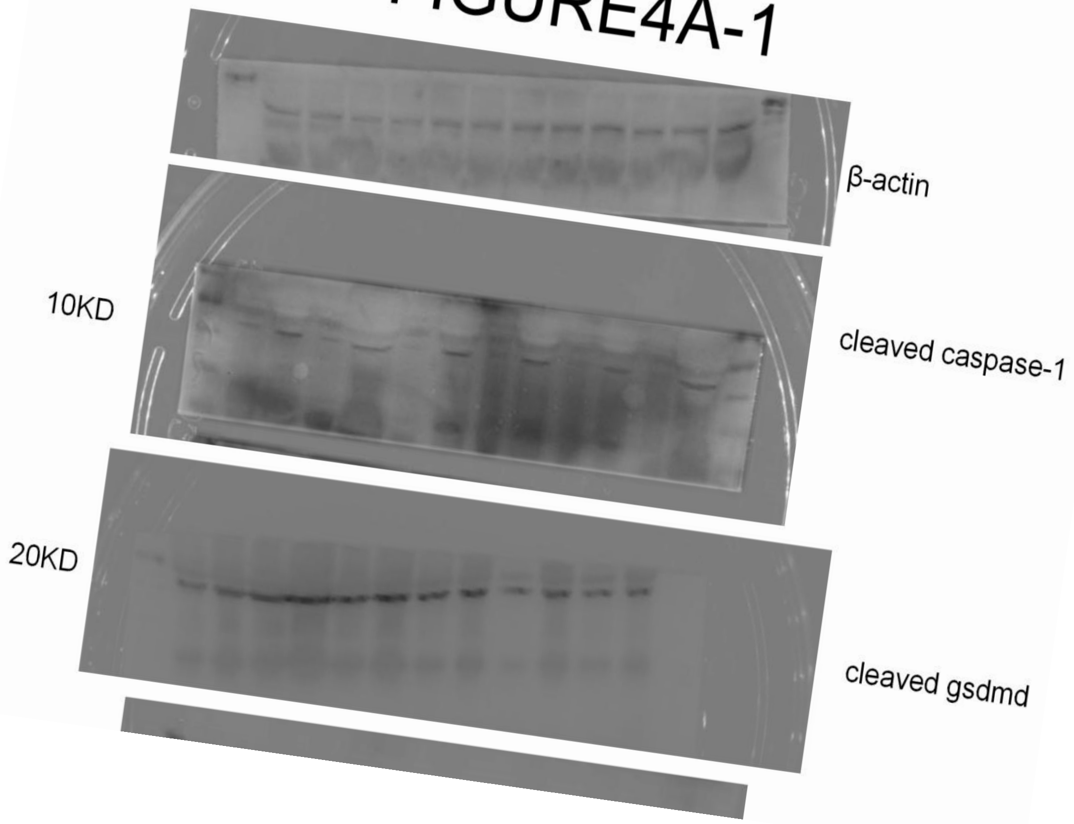

FIGURE4B

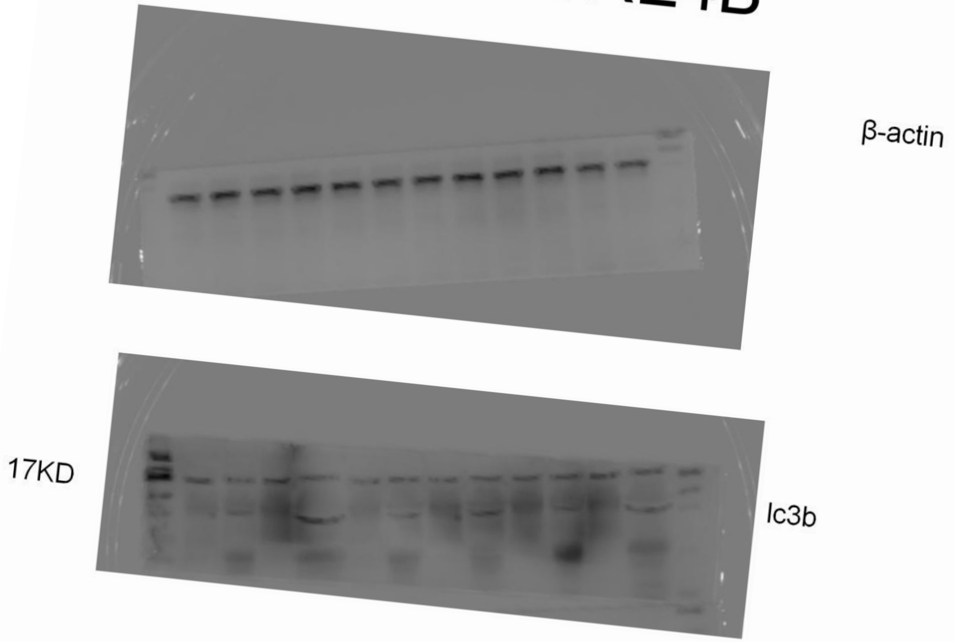

FIGURE4C

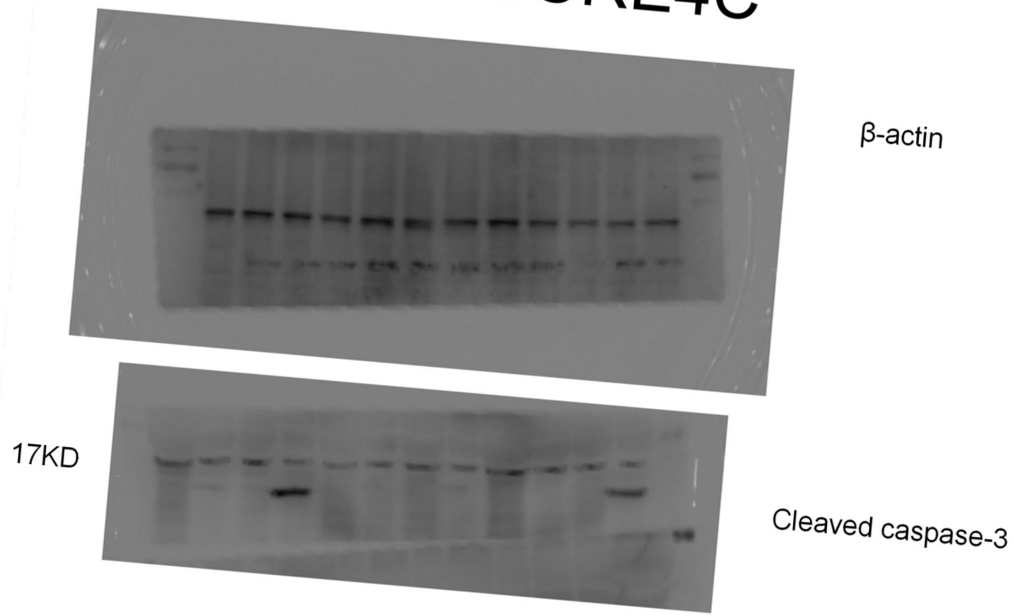

Supplement: Supplementary file 1 — Supplementary Material 1 [file 41598_2026_40893_MOESM1_ESM.pdf]
